# Potential Use of Emerging Technologies for Preservation of Rice Wine and Their Effects on Quality: Updated Review

**DOI:** 10.3389/fnut.2022.912504

**Published:** 2022-06-23

**Authors:** Jinjin Pei, Zhe Liu, Yigang Huang, Jingzhang Geng, Xinsheng Li, Sisitha Ramachandra, Amali Alahakoon Udeshika, Charles Brennan, Yanduo Tao

**Affiliations:** ^1^Shaanxi Key Laboratory of Bioresources, 2011 QinLing-Bashan Mountains Bioresources Comprehensive Development C. I. C., Qinba State Key Laboratory of Biological Resources and Ecological Environment, College of Bioscience and Bioengineering, Shaanxi University of Technology, Hanzhong, China; ^2^Northwest Institute of Plateau Biology, Chinese Acadamy of Science, Xining, China; ^3^School of Technology, Faculty of Engineering and Technology, Sri Lanka Technological Campus (SLTC), Padukka, Sri Lanka; ^4^Department of Biosystems Technology, Faculty of Technology, University of Sri Jayewardenepura, Nugegoda, Sri Lanka; ^5^Royal Melbourne Institute of Technology, Melbourne, VIC, Australia

**Keywords:** rice wine, fermentation, microbial safety, preservation, technologies

## Abstract

Rice wine, a critical fermented alcoholic beverage, has a considerable role in different cultures. It contains compounds that may have functional and nutritional health benefits. Bacteria, yeasts, and fungi commonly found in rice wines during fermentation can induce microbial spoilage and deterioration of the quality during its distribution and aging processes. It is possible to control the microbial population of rice wines using different preservation techniques that can ultimately improve their commercial shelf life. This paper reviews the potential techniques that can be used to preserve the microbial safety of rice wines while maintaining their quality attributes and further highlights the advantages and disadvantages of each technique.

## Introduction

Rice wine is a world-renowned fermented product that plays a crucial role in the wine culture of different countries. The production of alcoholic beverages based on rice fermentation has been practiced in many Asian countries for a long time. It is called “rice wine” because its alcohol content is close to that of wine, and its appearance is a clear and transparent light yellow liquid (1). Rice wine is known under different names based on the manufacturing procedures and raw materials used in different regions. In China (and throughout Asia), Shaoxing rice wine is a widely consumed type of rice-based fermented beverage (2). In the Republic of Korea, *makgeolli* is a traditional and trendy alcoholic drink (3). In Japan, *sake* is the most popular alcoholic beverage, and its consumption is assumed to contribute to a healthy life since it reduces the stress response.

Rice wine is made by fermenting refined rice at a low temperature of 9–11°C for 20–25 days. In the fermentation stage, the first batch of bacteria was mainly nitrate-reducing bacteria, and the second batch was *Leuconostoc mesenteroides varsake and Lactilactobacillus sakei* and yeasts. Because the pH of alkaline syrup is too high, which is not conducive to the good growth of yeast or producing good taste, it is also essential to control the number of lactic acid bacteria (1). These microorganisms also act as the source of microbial spoilage that occurs during transportation and can ultimately restrict the quality of rice wine (4, 5). Thermal processes, such as pasteurization, are widely utilized as sterilization techniques to improve the shelf life of rice wines. However, the heating of rice wine can also have a negative impact on its organoleptic properties, particularly the relationship between proteins, sugars and flavonoids. Non-thermal processing techniques can avoid these heat-induced quality changes, the addition of plant-based extracts such as seed extract from grapefruit and the addition of chitosan (5). This review summarizes recently published data on the potential technologies to preserve the microbial safety of rice wines and the positive or negative quality changes caused by each of these technologies in terms of the technological, sensory, and nutritional quality attributes. The development of rice wine preservation technology has been improving, and the processing methods described in some old documents need to be updated. We reviewed the literature after 2010, with rice wine, rice wine processing, rice wine preservation and rice wine quality as the key words. We searched the Google Academic, Web of Science, and Springer Nature databases as data sources.

## Functional and Spoilage Bacteria Present in Rice Wine

Bacteria can usually be classified as functional bacteria and spoilage bacteria. Functional bacteria refer to the strains responsible for producing enzymes and flavor compounds; spoilage bacteria are strains that cause food spoilage (6, 7).

During the manufacture of rice wine, the carbohydrate components of the rice are converted to sugars and then fermented by microbial elements (yeasts and molds) into alcohol.

**Figure 1:**
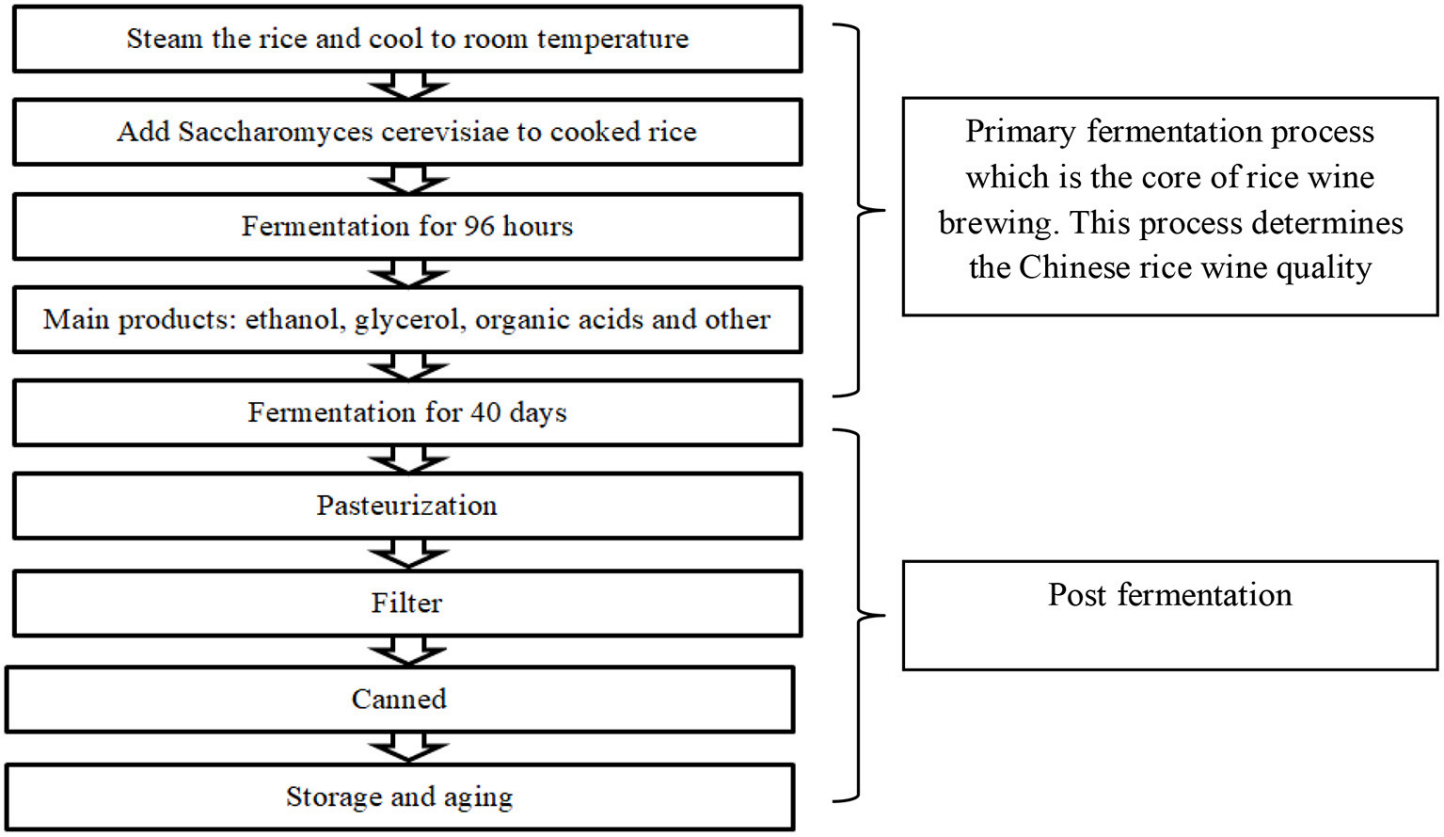
The basic fermentation process in Chinese wine preparation.

The general process of Chinese rice wine production is shown in Figure 1. In Chinese rice wine fermentation, wheat Qu may be used as a saccharifying agent with rice. The frequently used starter is yeast (*Saccharomyces cerevisiae*), facilitating simultaneous saccharification fermentation (8). *Nuruk* is a starter made of herbal extracts, and koji is used to produce Korean rice wine (*makgeolli*) (9).

Lv et al. (10) stated that bacteria are the primary microorganisms that are important in producing flavor-related compounds. It determines the overall acceptability of Chinese rice wines, whereas Nile (11) stated that the volatile compounds produced by yeasts are the leading cause of the organoleptic properties in *makgeolli* fermentation.

Fermentation with fungi and bacteria allows the saccharification of rice starch, which results in glucose, ethanol, and carbon dioxide production. Table 1 depicts the fermented alcoholic beverages from various countries and the primary starter cultures used in each country. Some microorganisms in the starter tend to remain active during bottling and dispensing (17). Suppose that any microorganisms remain in the alcoholic beverages after bottling. In that case, this could cause a food safety risk since these microorganisms can spoil and degrade the quality of the rice wine, and their control postfermentation is of considerable interest (18). Several pathogenic bacteria, such as *Bacillus cereus, Campylobacter jejuni, Staphylococcus aureus, Escherichia coli* 0157:H7 and *Salmonella* sp., can exist in rice wine (19–22). For example, the spoilage bacteria in Chinese rice wine mainly include *E. coli* and other foodborne pathogens, such as *Bacillus cereus, Campylobacter jejuni, Staphylococcus* aureus and *Salmonella sp*. The microorganism that causes the deterioration of Japanese *sake* is a bacterium called “hiochi”; Hiochi bacteria are generally composed of lactobacilli, namely, hiochi-lactobacilli and hiochi-bacilli. The former includes various kinds of lactic acid bacteria, including *Lacticaseibacillus casei, Lacticaseibacillus paracasei, Lacticaseibacillus rhamnosus, Limosilactobacillus fermentum, Lactiplantibacillus plantarum*, and *Lactobacillus hilgardii*. In contrast, the latter includes *Lactobacillus fructivorans and Lactobacillus homohiochi*. Because *hiochi-lactobacilli* are easily inactivated by pasteurization, it is considered that *hiochi-bacilli* are the actual spoilage bacteria of sake. The spoilage bacteria in *makgeolli* are *Escherichia coli, Bacillus cereus, Campylobacter jejuni, Clostridium perfringens, Escherichia coli* O157:H7*, Listeria monocytogenes, Salmonella sp., Staphylococcus aureus*, and *Yersinia enterocolitica* (12, 13, 23). Highly resistant endospores formed by *Bacillus cereus* are all active in the product or gastrointestinal tract. Keot et al. (14) detected the presence of harmful microorganisms, such as *Acidovorax, Herbaspirillum, Methylobacterium, Pantoea, Pseudomonas, Stenotrophomonas, Staphylococcus, Micrococcus, Acinetobacter, etc.*, in rice wine through Illumina technology. Kim et al. (19) suggested that *Bacillus cereus* contamination found during rice wine fermentation may come from rice and wheat flour. Kim et al. (19) stated that acetic acid bacteria promote the oxidation of sugars or ethanol and acetic acid production during rice wine fermentation. One of the main issues in the rice wine industry is finding effective techniques to preserve microbial safety while maintaining the nutritional and sensory attributes of the final products.

**Table 1:** Traditional rice wine in various countries, the starter cultures used in those rice wines, and the functional yeast and molds present in those starter cultures (12–16).

**Country**	**Type of rice wine**	**Functional yeast and mold present in starter cultures**
China	Mie-chiu, Shaohing, Huangjiu	*Saccharomyces cerevisiae, Wheat koji*
Japan	Sake	*A. Oryzae, A. awamorii, Saccharomyces cerevisiae,*
Korea	Yakju, Takju	*Yeast, Wheat barn koji, Nuruk (traditional natural starter with bacteria, yeast, and fungi)* *yeast, and fungi)*
India	Sonti	*Acidovorax, Herbaspirillum, Methylobacterium, Pantoea, Pseudomonas, Stenotrophomonas, Staphylococcus, Micrococ-* *cus, Acinetobacter*
Malaysia	Tapai	*Amylomyces rouxii, Rhizopus sp., Endomycopsis sp*.
Vietnam	Ruou Nep	*Mucor sp., Rhizopus sp., Aspergillus sp., Saccharomyces cerevisiae, Torulopsis candida*
Thailand	Sato, Krachae	*Mucor sp., Rhizopus sp., Candida sp., Saccharomyces sp*.
Phillipine	Tapuy	*Aspergillus sp., Endomycopsis sp., Hansenula sp., Rhodotorula glutinis, Candida parapsilosis*

## Effects of Plant Additives on the Preservation and Quality of Rice Wine

Increasing the health consciousness of consumers has prompted the use of natural food substitutes instead of synthetic food additives. Therefore, there is a growing demand for appropriate antimicrobial food additives that do not cause toxicity and maintain the sensory quality of fresh rice wines. In many countries, numerous locally available medicinal plants, herbs, and spices are added to the fermentation process of rice wine and rice beverages to inhibit the growth of harmful microorganisms in the final product. These plants can also provide specific nutrients for the starter cultures (24). The preservative effect of any spice and herb may be related to their composition of phytochemicals, antimicrobial compounds, and secondary metabolites, such as alkaloids and phenolics, which can modulate the growth of pathogenic bacteria (24). Herbs may help improve rice wine's sensory properties and give it some medical value (25). For instance, Murugan et al. (24) found that the fresh leaves and stems of the medicinal plants *Plumbagozeylanica* (Ceylon leadwort)*, Thelypteris clarkei C.F. Reed* (Lady fern)*, Clerodendrum D. Don* (Glory bower) *and Scoparia dulcis* (goat weed) resulted in elevated antimicrobial activity in fermented rice beverages and the inhibition of *Staphylococcus aureus i*n rice beverages. The presence of chemical constituents, such as flavonoids, alkaloids, tannins, carbohydrates, and glycosides, in these medicinal plants may have an antimicrobial effect (26). Zhou et al. (27) found that bamboo leaf extract had a significant effect on ethyl carbamate in compound microbially fermented rice wine. The addition of the primary leaf extract promoted the production of ethanol and amino acids in compound microbially fermented rice wine but inhibited the generation of aroma. Through sensory evaluation, it can be concluded that bamboo leaf extract can improve the comprehensive quality of rice wine.

In Northeast India, people add different plants and herbs to modulate the flavor of traditional rice wine. Some of these plants include *Albizia myriophylla* (Albezia) used in the state of Manipur, *Plumbago zeylanica* (leadwort), *Buddleja asiatica* (dog tail), *Gingiber officinale* (ginger) used in the state of Sikkim, and *Amomum aromaticum* used in Meghalaya. The Assam region also paid attention to the effect of adding banana (pineapple), jackfruit (jackfruit), *calotris gigantean* (giant milkeed), and Nagaland sprouted rice on rice wine. They found that bromelain, saponins, flavonoids, and polyphenols in pineapple are active compounds that control bacterial activity (28).

The physicochemical and microbial characteristics of *makgeolli* (Korean rice wines) incorporated with bananas have been investigated by Kim et al. (29) due to bananas' ability to add to the taste and nutritional value of the final product. Bananas also contain many secondary metabolites, such as phenolic compounds, which can act as antimicrobials.

Choi et al. (30) found that grape seed extracts influence the growth of both the brewing and spoilage of microorganisms in *makgeolli*. *A* concentration of 0.1–0.2% grape seed extract has been reported to prolong the shelf life of bottled fresh *makgeolli* by decreasing the concentration of bacteria and yeast. In addition, grapefruit seed extract has been reported to possess natural antimicrobial properties due to antimicrobial compounds, including naringin, limonoid, quercetin, kaempferol, and citric acid. These seed extracts do not negatively affect rice wine color, smell, or overall acceptance (31).

Jeong et al. (3) found that *makgeolli* had the best antioxidant activity when 0.45% steaming and drying deodeok *(Codonopsis lanceolata)* was added. In general, plant extract additive is a natural and healthy preservative for rice wine, and it can also make rice wine obtain some beneficial substances attached to plants. However, plant additives often affect fermentation strains, resulting in a decline in rice wine quality. Improving the shelf life of rice wine and reducing the impact on the quality of rice wine are also problems to be solved in the future.

Table 2 illustrates the effects of a few plant-based and nonplant-based natural additives that have been used in different rice wines.

**Table 2:** Effect of different plant-based and nonplant natural additives on the quality attributes of rice wines (3, 9, 26, 32, 33).

**Rice wine type**	**Natural additives used**	**Quality characteristics**	**References**
**Plant-based additives**			
Korean Takju	Natural honey (5%)	• Temperature, pH, acidity, and total sugar content showed no significant differences • Increase the flavor	Jung et al. (32)
Korean Takju	Codonopsis lanceolate (0,0.15%,0.3%,0.45%)	• The content of alcohol and polyphenols increased, while the content of reducing sugar decreased	Jeong et al. (3)
Chinese yellow rice wine	Bamboo leaves extract	• Enhanced antioxidant activity • Inhibit arginine metabolism • Prevent the reaction of urea and citrulline with ethanol	Zhou et al. (26)
Korean Yakju	Lotus leaves	• Lotus-leaf Yakjus containing fresh leaves and dried leaves were preferred to the others in color and flavor among Yakjus prepared with lotus leaves before cold storage.	Choi et al. (29)
**Non-plant based additives**			
Takju	0.001% of Chitosan	• Moderately decrease the viable microbial cells and yeasts • More stabilized in terms of turbidity during storage at 10°C for 12 days compared to untreated wine • Improved the sensory qualities compared to untreated wine	Kim et al. (33)
Makgeolli	Lysozyme 270ppm and glycine 0.27%, or Lysozyme 450 ppm and glycine 0.45%	• Suppressed the acid formation of wine • Increased the nucleotides contents of inosine monophosphate and inosine content • Improved the taste and palatability	Lee and Ahn (17)

## Effect of Heat Treatment on the Preservation and Quality of Rice Wine

Thermal processes are well known as food preservation methods (34). These techniques are economically feasible and efficient for inactivating pathogenic microorganisms in rice wines. In some countries, commercial rice wine is distributed in two forms (pasteurized or unpasteurized). Rather than using high-temperature short-term pasteurization (80°C for 23 secs), rice wines (such as *makgeolli*) are often treated using low-temperature, long-term pasteurization. Low-temperature, long-term pasteurization (55–65°C for 10–15 mins) is reported to cause some issues in sensory attributes, such as a strong off-flavor that gives a burnt odor, a reduction in color, and a separation of the layers in the final product. Such treatments can result in a commercially stable shelf life of 14 to 50 days (35). However, high-temperature processing can cause a reduction in color and sedimentation and can produce a strong off-flavor. Thus, food manufacturers prefer to use the low-temperature long-term process.

Park et al. (35) reported changes in the volatile components of unpasteurized and pasteurized *makgeolli* during storage (30 days). They found that the odor-related compounds in pasteurized rice wine were not changed over the storage period compared to those in non-pasteurized rice wine. However, Li et al. (21) stated that high-temperature (75, 80, 85, 90, 95°C) sterilization affected the sugar content of rice wine, which is considered an important quality indicator.

Kim et al. (19) compared commercially available sterilized Korean rice wine (*makgeolli*) from different plants subjected to different temperature/time combinations for sterilization. They stated that rice wines subjected to high-temperature sterilization (95°C/ <1 min) had the most significant bactericidal effect of decreasing the aerobic bacterial counts and the microbial numbers of *Clostridium jejuni, Clostridium perfringens, Escherichia coli 0157:H7, Listeria monocytogenes*, and *Staphylococcus aureus*. In addition, these treatment conditions were reported to either eliminate or significantly reduce the lactic acid bacteria, fungi, and acetic acid bacteria. The researchers reported that even with sterilization of wine at 85°C/1 min followed by 85°C/2 min, *Bacillus cereus* spores were present, but not the vegetative cells. Jeon (36) also reported the presence of *B. cereus* in 61% of sterilized products tested in Korean *makgeolli* and *yakju*. Lv et al. (13) illustrated the presence of *Bacillus cereus* cells in Chinese rice wine, increasing the heat treatment time to 60, 70, and 80°C, leading to a reduction in their value.

The most appropriate strategy to control *B. cereus* in rice wine would be to prevent the contamination of the rice wine rather than striving to eliminate the spores at a later stage (19). The processing factors (pH, water activity) can determine the heat resistance of the microorganisms. However, the duration of the exposure and temperature have a major impact on heat resistance (37). The medium's proteins and sugars act as a protective barrier to heat damage, while low water activity also provides protection (38). As Lv et al. (13) observed, the presence of protein and glucose in Chinese rice wines reduced the thermal sensitivity of *Bacillus cereus*. In contrast, the medium-high alcohol content and acidic pH increased the lethality. This could be attributed to disruption of the microbial cell wall due to the acidic pH and alcohol contents.

The other widely used thermal treatment for rice wines is conventional boiling (80–95°C for 15–30 min), which is considered a thermal sterilization method to kill bacteria, fungi, and other microorganisms. However, the drawbacks of this technique include the negative effect of temperature on the flavor characteristics, the low viscosity of the liquid, and the high energy usage, which limits the expansion of this method on an industrial scale (39).

It has been reported that conventional boiling has a bactericidal effect in the preparation of different rice wine types. Yang et al. (40) stated that thermal processing (90°C/15 min) could eliminate 99% of the microorganisms in rice wines. These authors further reported that conventional boiling at 90°C/15 min causes an unavoidable loss of volatile alcohols and aldehydes in Chinese rice wines, resulting in overall low acidity. In addition, this study showed that thermal treatment decreased the ethanol content in rice wine compared with non-treated wine due to the oxidation and esterification reactions caused by the thermal treatment. Thermal treatment reduces sugar content due to the Millard reaction, which reacts with sugar and free amino acids (41). In terms of the sensory characteristics of the rice wine, thermal boiling increased the loss of red pigments, and it tended to deteriorate the flavor.

According to the research conducted thus far, different sterilization parameters should be selected because different rice wine components and microbial survival rates are different to prevent adverse effects on the quality of rice wine. However, very few studies are available on determining the effects of rice wine composition on sterilization and the fundamental mechanisms of heat inactivation under different environments. For instance, Wu et al. (42) showed that Chinese rice wine should be sterilized at 85–95°C, as these rice wines have relatively high amounts of protein and the concentrations of sugar vary. Under certain conditions, the heating temperature should be carefully chosen to prevent melanoidin development from sugars and amino acids. For example, a Chinese rice wine that has been boiled at higher temperatures (90–95°C) will have a sugar content lower than 15.0 g/L, whereas semisweet and sweet Chinese rice wine that has a sugar content that is >40.0 g/L will have been boiled at 85–90°C (42, 43). Under the condition of heat sterilization, spores still exist in rice wine, which has always been a problem. However, achieving the maximum lethality of pathogenic microorganisms through excessive heating will reduce the sensory properties of yellow rice wine (21, 44). Therefore, either minimizing the intensity of the thermal treatment and the duration of exposure or substituting the thermal treatment with an appropriate non-thermal technology would be appropriate to preserve the quality without compromising safety (13).

## Effects of Ultrahigh Temperature (UHT) on the Preservation and Quality of Rice Wine

Ultrahigh-temperature (UHT) processing uses continuous-flow thermal processing for the sterilization process (45). UHT operates at ~130–150°C for a short duration to reduce microbial loading (46). Therefore, the advantage of UHT is to use a shorter processing time to retain the natural sensory and nutritional characteristics of food and reduce the loss of quality. Jin et al. (47) stated that UHT treatment (125°C/5 s) inactivated the yeast and total bacteria in Korean rice wines (*makgeolli*) while having no adverse effects on the pH, titratable acidity, or sugar contents of the rice wine during storage (at 15°C). However, the drawback was an increase in off-flavor development with an increase in storage time, which might be attributed to sulfur-containing amino acid degradation by UHT treatment.

Yang et al. (40) also reported that UHT (125°C/5 s or 135°C/5 s) could eliminate the microorganisms in Chinese rice wines, resulting in above 99% lethality. Concerning the sensory and other quality attributes, the UHT treatment increased the total acidity of the rice wines after treatment. According to Tian et al. (48) this increase could be attributed to the production of aldehydes due to alcohol oxidation. These aldehydes later oxidize to acids, which tend to increase the total acidity of the rice wine. In addition, the UHT increased the Millard reaction in the rice wines, which caused a reduction in the sugar content of the final product. However, the UHT did affect the flavor of the rice wine with the short processing time (5 s). Therefore, ensuring the flavor of rice wine is one of the complex problems to be solved when UHT eliminates spoilage bacteria.

## Effects of High Hydrostatic Pressure Processing (HHP) on the Preservation and Quality of Rice Wine

Consumer demand for healthy and nutritious food has driven people to seek new food processing alternatives. It is expected that the use of these new techniques may produce safer food while retaining fresh-like quality, even though a few challenges still exist. The high hydrostatic pressure used in a refrigeration room or at a moderate temperature is believed to reduce/inactivate spoilage and pathogenic bacteria in the food. Compared to conventional heating, HHP prevents negative consequences on the quality of rice wine (49). The shorter processing time that HHP technology requires would be useful to maintain the quality of the food products in contrast to the foods treated by conventional methods. The main principle of the HHP treatment involves subjecting the food to water pressure (100–900 MPa) by placing the packaged food inside a pressure vessel. Rendueles et al. (50) stated that microbial inactivation by HHP is caused by changes in the cellular functions and the integrity of the microflora membrane. Cell death of the microflora is believed to occur due to any moderations in ion exchange, changes in the fatty acid composition, denaturation of cell proteins, or processes affecting enzyme activity. Several studies have observed that HHP can decrease the initial numbers of bacteria, yeasts, and mold in rice wine. Buzrul (51) found that HHP at 500 MPa pressure for ≤30 min has antimicrobial effects on rice wine. Xu et al. (15) found that adding calcium and magnesium ions during extrusion can give yellow rice wine a high phenol content and antioxidant activity. Jin et al. (47) declared that the HHP treatment (400 MPa for 10 min) of Korean rice wine (*makgeolli*) resulted in a reduction in total bacterial, lactic acid bacteria and yeast counts in sterilized *makgeolli* during storage. Yang et al. (40) also reported that HHP treatment (200, 400, and 600 MPa for 10 and 20 min) could eliminate the total aerobic bacteria in rice wines, and the lethal rate was reported to be > 99%. Ha et al. (52) illustrated that lactic acid bacteria and yeast were not observed immediately after HHP at 400 MPa for 5 min because they were decreased under the detection limit. However, this study further found white wrinkly shaped bacteria in the samples even after HHP treatment, confirmed as *Bacillus amyloliquefaciens*. These findings suggest that although HHP inactivates most of the bacterial growth conditions that support the growth of Bacillus, Bacillus can still compete with other strains. Bañuelos et al. (53) noted that ultrahigh-pressure sterilization can eliminate local microorganisms in grape juice and effectively inactivate oxidase, reducing browning and improving sensory quality.

However, it is crucial to select the best HHP treatment conditions that can positively impact the sensory attributes of rice wines. The study of Jin et al. (47) which used HHP treatment at 400 MPa for 10 min for *makgeolli*, maintained pH stability and total acidity during storage (15°C/20 weeks). In contrast, those with untreated *makgeolli* decreased over time. Apart from that, HHP could maintain a desirable color, flavor, taste, and overall sensory qualities in rice during storage. Yang et al. (40) reported that HHP treatment increased the reducing sugar content of rice wines, which might be due to the increased activity of the enzymes, and the activation of the Saccharomyces enzymes as a result of the HHP treatment may be the factor that affects reducing the sugars overall. HHP also increased the free amino acid contents due to the proteolysis promoted by HHP. Even though HHP may promote the Maillard reaction, the speed is lower than that of proteolysis. Moreover, the HHP treatment (>200 MPa) resulted in more flavor compounds than the non-HHP-treated rice wine. However, the pressure and time involved in the HHP treatment play a vital role, suggesting 600 MPa/10 min, which accelerates the oxidation and esterification processes in the rice wines. At the same time, 600 MPa is excessive for the rice wine flavor in this study.

Ha et al. (52) showed that 400 MPa HHP treatment promoted an increase in reducing sugars in *makgeolli*, while the reducing sugar content of *makgeolli* not treated with HHP decreased. The researchers examined *makgeolli*, which was treated with HHP and stored at 25 °C for 6 days, and found that HHP could slow the increase in alcohol content, pH and acidity. The research shows that HPP can prolong the shelf life of *makgeolli* by inactivating lactic acid bacteria and yeast.

Tian et al. (48) showed that as the storage time (at 10–15°C for 18 months) increased, HHP-treated (200/550 MPa for 30 min) hongqu rice wines showed a significant difference in some oenological properties, such as alcohol % and total solids. This study noted that the alcohol content in the HHP-treated rice wine rapidly decreased after 6 months of storage. The highest-pressure condition of 550 MPa tested in this study had a more rapid decrease in alcohol than the 200 MPa. Additionally, the 550 MPa treatment led to a rapid decrease in total solids after 3 months of storage due to the acceleration of sugar-amine condensation by the HHP treatment and a significant decrease in total free amino acids. This study showed that the Maillard and oxidation reactions in the HHP-treated wines were promoted due to the fast reduction in the total solid content, the reduced free amino acids, and the higher content of the ketones.

Therefore, rice wines treated with HHP may not be appropriate for long-term storage under certain conditions. HHP treatment can quickly deteriorate the sensory quality of wine, producing a higher bitterness and higher astringency level (48). Considering the above facts, it can be suggested to use only certain pressure conditions to facilitate maximum microbial safety while preserving nutritional and sensory attributes. Therefore, the problem of temporary storage after HHP treatment should be optimized in future research to obtain the maximum sensory and nutritional effect in any type of rice wine.

## Effects of Pulsed Electric Fields (PEFS) on the Preservation and Quality of Rice Wine

Pulsed electric field processing is an emerging non-thermal food technology that can be used to treat liquid foods, such as fruit juices and rice wines (54, 55).

Pulsed electric field (PEF) technology is a short-term electric treatment that works (ns-ms) promptly with electric field strengths ranging from 100 to 300 V/cm to 20–80 kV/cm. At high electric fields (> 20 kV/cm), the PEF can act as a substitute for conventional thermal processing to inactivate pathogenic bacteria and enzymes. PEF has an advantage over traditional thermal processing because it can retain the sensory, nutritional, or health-promoting ingredients of liquid food products (56).

Recently, PEF technology has been explored as a preservation technology to eliminate spoilage bacteria in wines. A few studies have been conducted to test whether PEF technology can control spoilage in the microflora in grape wine and beer (57). However, the inactivation efficacy of different microbial species by PEF treatment is primarily related to their different susceptibility levels. Therefore, the inactivation efficiency depends on the genetic characteristics of the specific microorganism (58).

Huang et al. (54) used PEF to control the spoilage microorganisms in Chinese rice wines. PEF was applied at an electric field strength of 12–21 kV/cm (treatment duration 30–180 μs, monopolar square pulses) to investigate the inactivation efficiency of PEF to inactivate the spoilage yeast *Saccharomyces cerevisiae* in this study. The decrease in the number of yeasts has been supported by finding the PEF-induced destruction of the cell membrane structure. The highest inactivation was observed with PEF at 21 kV/cm and 180 μs, suggesting that the high intensity of the PEF causes higher inactivation. Thus, the complete inactivation of the microorganisms could be achieved with PEF at an adequate treatment intensity. However, for each microorganism, the optimum conditions need to be found. Therefore, the required amount of energy could be calculated without wasting too much unwanted energy and affecting the quality of the rice wine.

However, the efficiency of microbial inactivation via PEF treatment is also closely related to the treatment temperature. Microbial sensitivity to PEF increases with increasing temperature. Huang et al. (54) illustrated that PEF applied at different temperatures had a different antimicrobial potency. This study further showed a higher inactivation rate (2.05 log-cycles) of the yeasts in the Chinese rice wine that was treated at a higher initial temperature for the PEF treatment (18 kV/cm, 150 ms at 25°C).

The lethality is also based on the composition of the rice wine, basically, the concentration of ethanol. Puertolas et al. (59) stated that the lethal influence of PEF treatment on the microbes in rice wine is increased due to the high ethanol concentration present in the wine. Moreover, Milani et al. (60)investigated the impact of PEF treatment (45 kV/cm, 46.3 pulses, and 70 ms) on the inactivation of *Saccharomyces cerevisiae* spores in beer and found that beer samples with the highest log reduction had the highest alcohol concentration. These results suggested that the use of PEF for samples with a high alcohol concentration can result in higher microbial inactivation.

Apparently, PEF processing does not have a considerable negative effect on rice wine sensory and other quality attributes. Shen et al. (61) showed that micro oxygen combined with electric field treatment could greatly improve the flavor intensity of yellow rice wine. Huang et al. (54) found that PEF treatment at 12–21 kV/cm did not cause changes in the soluble solids, pH, or color during the post-PEF treatment period or storage at 22°C. However, finding treatment conditions suitable for most microorganisms is also a complicated problem that must be overcome in the future. Further investigation is needed to study the feasibility of the use of PEF in microbial inactivation before promoting the use of PEF on a commercial scale.

## Effects of UV-C Irradiation and Electron Beam Irradiation (EB) on the Preservation and Quality of Yellow Rice Wine

Ultraviolet (UV) irradiation has been used to process liquid foods (34). It is used as a substitute for thermal processing. Reducing microbial corruption will have a negative impact on the sensory characteristics of liquid food. UV radiation technology can inhibit the reproductive function of other cells by destroying the DNA of microorganisms. The penetration and effectiveness of UV-C depend on the absorbance, density, color, soluble solids and density of beverages (62).

The principle of electric beam irradiation (EBI) is the generation of electrons from a cathode using commercial electricity in a vacuum environment. These electrons are subsequently emitted from an electron gun in sequence, creating a beam of electrons (63). EBI can directly or indirectly damage the metabolism and cell chemical reactions of microorganisms or cause consequent microbial cell death (63).

However, based on a review of the literature, only a few studies are available on the use of UV-C and EBI to enhance the microbial safety or sensory quality of rice wines. Kim et al. (29) showed the impact of UV-C and EBI on the different quality attributes of the *makgeolli* during storage (at 4°C for 15 days). This investigation stated that EBI (at 0.5, 1, 2, and 3 kGy) effectively controlled the microorganisms in the *makgeolli* compared with the UV-C treatment. However, there are certain drawbacks involved with using both of these treatments on the quality of the *makgeolli* related to an increase in the pH of the wine over the storage time. EBI tended to have a higher acidity in the wine than UV-C-treated rice wine. However, further studies are recommended to determine how to minimize the quality deterioration in rice wines during storage after UV-C or EBI treatment.

Jin et al. (47) reported that UV sterilization (15 W germicidal lamp for 10 min) completely inactivated the yeasts and the total bacteria in *makgeolli*. This treatment did not significantly affect the pH or the titratable acidity, nor did it reduce the sugar content during storage (at 15°C for 20 weeks). This might be attributed to the inactivation of the majority of the microorganisms by sterilization. Therefore, the pH and the titratable acidity of the wines are not affected. On the other hand, this study showed that UV treatment could maintain the color of *makgeolli* during storage. To use irradiation as an alternative approach for thermal treatments, further studies on the effect of irradiation at different doses on various spoilage microorganisms should be conducted while directing attention to the other quality attributes. In addition, the available information on UV-C or EB radiation is not enough to conclude that the potential application of these technologies in commercial rice wine production will be beneficial.

## Effect of Nanomaterials on the Preservation of Rice Wine

In recent years, nanotechnology has developed rapidly in the food industry and can be used in food production, packaging and transportation. The application of nanomaterials in food preservation can improve the microbial pollution of food and improve the bioavailability of nutrients. Moreover, aerosols using water nanostructures can also kill foodborne pathogens such as *Salmonella aureus, Escherichia coli*, and *Bacillus atrophaeus* (64). The unique physical, chemical, and biological properties of nanoparticles disrupt enzyme activity and destroy intracellular organelles. Therefore, metal oxide nanoparticles have attracted much attention as antibacterial agents. According to González-Arenzana et al. (58) silver nanoparticles have an inhibitory effect on all microbial populations in the wine used for testing. Before adding sulfur dioxide and silver nanoparticles, the author analyzed the white wine samples collected after 1 day and 30 days, respectively, and then analyzed the red wine polluted by Breton for 15, 30, and 60 days. Finally, the results show that silver nanoparticles are used as antibacterial agents and have more potential than SO_2_ in controlling lactic acid bacteria (LAB), acetic acid bacteria (AAB), and yeast. However, the safety of nanosilver ions after entering the human body is not apparent. Pachnowska et al. (16) believe that silica nanospheres can replace sulfur dioxide to stabilize microorganisms in wine. It is worth noting that the author's experiment is tested on one strain. This experiment is in the laboratory stage. Whether it is effective for other bacteria must be further studied. In short, nano preservation technology has become a hot topic in the food industry in recent years; however, its application in rice wine preservation is slightly scarce, and whether nanomaterials, such as nanosilver ions, nano silicon and nano clay, are harmful to the human body needs further research.

## Conclusion and Future Perspectives

Even though several sterilization methods can be used for food processing, each method has specific opportunities and drawbacks. Therefore, the most appropriate method should be selected according to the sterilization purpose and food. For example, heat treatment is widely used to preserve rice wine worldwide. However, some studies still show that due to the specific adverse effects on the final sensory quality of rice wine, the deterioration of the quality of rice wine should be limited during these heat treatments. Although heat treatment has clarified its beneficial role in protecting the microbial safety of products, commercial rice wine producers are still looking for the most effective choice to obtain nutritionally and sensorily acceptable rice wine. Therefore, the primary concern of the rice wine industry is to find an effective process to ensure the microbial safety of rice wine and minimize quality defects. For example, compared with pasteurization, plant additives not only do not reduce the sensory quality of rice wine but also bring some unique flavor to plants. However, we need to further explore the safety of plant additives and their impact on the fermentation process. UHT can kill some bacteria that cannot be killed by pasteurization in terms of the sterilization effect, but its impact on product flavor is also difficult to solve. Compared with pasteurization and UHT, HHP can well ensure the sensory quality of rice wine, but the problem that rice wine cannot be preserved for a long time is highlighted. Compared with the above sterilization methods, PEF and UV-C irradiation and electron beam irradiation (EB) technology can package the sensory quality of rice wine and prolong its storage period. However, PEF requires different processing conditions for different microorganisms and obviously cannot be used in factories. Finding a method suitable for most microorganisms is a complex problem that must be overcome. Nanotechnology is one of the rapidly developing food preservation, transportation and processing technologies in recent years, but its application in rice wine is little known, and we need to explore it.

Emerging technologies, such as plant additives, HHP, UHT and pulsed electric field treatment, can be effectively used to produce commercial yellow rice wine. However, further research is needed to immediately eliminate different spoilage microorganisms in rice wine after treatment and during storage at different temperatures. In addition, before implementing these processes in the yellow rice wine industry, more research is needed to determine the safety of these compounds and the toxicity problems related to different technologies and their impact on human health.

## Author Contributions

JP: conceptualization, design of the work, visualization, resources, funding acquisition, and writing—review and editing. ZL and YH: acquisition, resources, investigation, and formal analysis. JG, XL, and SR: supervision, conception or design of the work, resources, and investigation. AA: data acquisition, formal analysis, conceptualization, and writing—review and editing. CB and YT: supervision and review. All authors contributed to the article and approved the submitted version.

## Funding

This study was funded by a Special Support Plan for High-Level Talents in Shaanxi Province (For JP), Open Project Program of Shaanxi Key Laboratory of Bio-resources (SLGPT2019KF03-04), Qin-Ba Key Laboratory of Biological Resources and Ecological Environment (cultivation) Program (SXC-2106), the Foreign Expert Project of the Ministry of Science and Technology (G2021041001, DL2021041001, and QN2021041001), and the Research Project of Shaanxi Provincial Department of Science and Technology (2021JZY003).

## Conflict of Interest

The authors declare that the research was conducted in the absence of any commercial or financial relationships that could be construed as a potential conflict of interest.

## Publisher's Note

All claims expressed in this article are solely those of the authors and do not necessarily represent those of their affiliated organizations, or those of the publisher, the editors and the reviewers. Any product that may be evaluated in this article, or claim that may be made by its manufacturer, is not guaranteed or endorsed by the publisher.
